# Reducing Peritoneal Cell Dissemination in Laparoscopic Uterine Surgery: A Comparative Pilot Study on Morcellation Techniques and Peritoneal Irrigation

**DOI:** 10.3390/jcm14103383

**Published:** 2025-05-13

**Authors:** Lorenz Kuessel, Lejla Sandrieser, Gerda Hofstetter, Florian Heinzl, Michal Mara, Adéla Richtárová, Eliana Montanari, René Wenzl, Alexandra Perricos-Hess, Heinrich Husslein

**Affiliations:** 1 Department of Obstetrics and Gynecology, Medical University of Vienna, Währinger Gürtel 18-20, 1090 Vienna, Austria; 2 Department of Gynecology, Obstetrics and Neonatology, Charles University of Medicine in Prague, Katerinská 32, CZ-121 08 Prague, Czech Republic

**Keywords:** power morcellation, manual morcellation, cell spread, irrigation, fibroids, laparoscopic hysterectomy, myomectomy, minimally invasive surgery, occult malignancy

## Abstract

Following the U.S. Food and Drug Administration’s warning against power morcellators due to potential cell dissemination of occult malignancy, there has been a shift away from minimally invasive approaches. This concern also overshadows the well-documented advantages of minimally invasive surgery in benign gynecology. **Objectives**: To evaluate whether intraperitoneal cell dissemination during laparoscopic surgery for uterine fibroids can be reduced by (i) the choice of morcellation method and/or (ii) copious irrigation after the procedure. **Methods:** This prospective multicenter comparative pilot study included 72 women undergoing laparoscopic myomectomy (LM) or total laparoscopic hysterectomy (TLH) for benign conditions. Women were divided into four groups in order to compare different types of morcellation, including a reference group without morcellation: (i) LM with power morcellation (*n* = 21, Group A), (ii) TLH with en-bloc transvaginal tissue removal without morcellation (*n* = 17, Group B), (iii) TLH with manual vaginal morcellation (*n* = 19, Group C), and (iv) TLH with contained manual vaginal morcellation (*n* = 15, Group D). Patients receiving cold knife morcellation were randomized into Groups C or D. In order to assess cell spread before surgery, after surgery but before morcellation, after morcellation, and after abdominal irrigation with a total of 3000 mL saline solution, peritoneal washings were collected at six timepoints. **Results:** After specimen removal (TP3), cell spread was significantly higher in cases with power morcellation [13/19 (68%) in Group A] compared to transvaginal cold knife morcellation, both contained and uncontained [Group C 1/14 (7%) and Group D 1/19 (9%)] (*p* < 0.001), or to TLH with en bloc removal [Group B 1/17 (6%)]. Saline irrigation reduced the positive cytologies. After 3000 mL (TP6), the difference between Group A and the TLH groups was not significant [4/18 (22%) vs. 3/45 (7%), *p* = 0.079]. **Conclusions:** Our study shows that (i) transvaginal cold knife morcellation results in significantly less peritoneal cell dissemination than power morcellation, and (ii) peritoneal irrigation with 3000 mL of saline significantly reduces residual cell presence. These findings could support maintaining minimally invasive approaches while addressing safety concerns.

## 1. Introduction

Over recent decades, minimally invasive surgery has gained popularity due to its advantages in reducing intraoperative blood loss, postoperative pain, and length of hospital stay compared to laparotomy [1]. The introduction of power morcellators, enabling tissue removal through small incisions, was pivotal in laparoscopic fibroid surgery and laparoscopic hysterectomy for large uteri [2]. However, power morcellation poses risks of intra-abdominal cell spread, potentially leading to complications such as parasitic leiomyomas, iatrogenic endometriosis, or worsening oncological outcomes in cases of occult malignancy [3,4]. In 2014, the FDA issued a warning against power morcellation in myomectomy and hysterectomy due to concerns about spreading unsuspected leiomyosarcoma [3], resulting in decreased use of minimally invasive techniques and increased major surgical complications and hospital readmissions [4].

Since then, new surgical instruments, including laparoscopic containment bags, advanced morcellators, and new minimally invasive surgical techniques like the application of cold knife morcellation through mini-laparotomy, have been developed to mitigate these risks [5,6,7,8]. However, studies show that fibroid cells can spread during enucleation, even before morcellation, rendering containment insufficient to prevent cell dissemination [9,10]. Yu et al. explored an alternative by using copious intraoperative irrigation and suctioning, finding that 3000 mL of saline or sterile water significantly reduced cell spread after power morcellation in a small pilot study [11].

Building on this, we conducted a larger investigation to assess smooth muscle cell presence in peritoneal washings before and after different types of morcellation, i.e., power morcellation and manual transvaginal cold knife morcellation with or without a containment bag, as well as after total laparoscopic hysterectomy with vaginal en bloc tissue removal as a reference group. Furthermore, we examined the effect of saline irrigation in reducing cell spread after morcellation.

## 2. Materials and Methods

### 2.1. Study Population

In this prospective comparative pilot study, women undergoing laparoscopic myomectomy or total laparoscopic hysterectomy (TLH) for benign indications such as uterine fibromas, adenomyosis, and heavy menstrual bleeding were asked to participate during surgical planning.

At the time of surgical planning, all patients underwent a comprehensive preoperative evaluation, including transvaginal ultrasound, performed by experienced gynecologic sonographers. In cases where imaging findings or medical history raised suspicion for malignancy, patients were referred to the institutional gynecologic oncology tumor board for further assessment and were excluded from this study. The uncertainty in the definitive exclusion of malignancy in patients with presumed fibroids and its implications were discussed thoroughly with all patients during the informed consent process.

Surgery was performed at the Departments for Gynecology and Obstetrics at Medical Universities Vienna and Prague between December 2019 and August 2022. Exclusion criteria were age < 18 years, myomas amenable to hysteroscopic resection, preoperative suspicion of malignancy, and intraoperative conversion to laparotomy.

The study was approved by the ethics committee of the Medical University of Vienna (EC Nr. 1774/2018) and Charles University in Prague (EC Nr. 219/20 S-IV). Written informed consent was obtained from each patient before surgery, and the patients’ baseline characteristics were prospectively collected. Information regarding the patients’ baseline characteristics as well as surgery and morcellation type was collected using CRF and the operative report.

During myomectomies, an incision was made on the surface of the fibroid using monopolar energy, the fibroid was grasped, vascular attachments between the myometrium and the fibroid were coagulated using bipolar energy, and the fibroid was enucleated with blunt dissection. The myometrial defect was closed using a self-absorbable multilayer running suture. The fibroids were then power morcellated using a Nouvag morcellator-type TCM 3000 BL.

In patients undergoing TLH, all specimens were removed vaginally. Morcellation was performed using a cold knife with or without a specimen bag. Women in the TLH group requiring morcellation based on preoperative examination, including transvaginal ultrasound, were randomly assigned to cold knife morcellation with or without a specimen bag. Randomization was performed on the day of surgery according to the next available number in the concealed sequence of a computer-generated randomization plan. All specimens were sent for routine histopathological analysis.

In order to establish a uniform workflow across both centers and thereby eliminate potential confounders within the framework of the multicenter study, reciprocal site visits were conducted at the outset of the study, and surgical procedures and measures were rigorously standardized.

### 2.2. Study Groups and Sample Collection

The following study groups were created for this study in order to compare different types of morcellation, including a reference group without morcellation: women undergoing (i) laparoscopic myomectomy with power morcellation (*n* = 21, Group A), (ii) TLH with en bloc transvaginal tissue removal without morcellation (*n* = 17, Group B), (iii) TLH with manual vaginal morcellation (*n* = 19, Group C), and (iv) TLH with contained manual vaginal morcellation using a contained extraction system (Alexis O Wound Protector-Retractor and Specimen Containment Bag, Applied Medical, CA 92688) (*n* = 15, Group D). A minimum of 15 patients per group was planned according to the study protocol.

In order to assess cell spread before the surgery started, after the surgery but before morcellation, after morcellation, and after abdominal irrigation with a total of 3000 mL saline solution, peritoneal washings were obtained during the procedures at six prespecified timepoints (TPs): after trocar insertion and creation of a pneumoperitoneum, before performing the myomectomy or hysterectomy (TP1), immediately after myomectomy or hysterectomy, before morcellation of the specimen (TP2), after the removal of the specimen (en bloc or after morcellation) and all visible tissue fragments (TP3), after irrigation and suctioning with 1000 mL saline solution (TP4), after irrigation and suctioning with another 1000 mL (i.e., total irrigation with 2000 mL) saline solution (TP5), and after irrigation and suctioning with an ultimate 1000 mL (i.e., total irrigation with 3000 mL) saline solution (TP6). Peritoneal washings were performed using a 100 cc saline solution that was instilled and removed using a 100 cc syringe and a reusable laparoscopic suction irrigator. For each washing, a new reusable laparoscopic suction irrigator system was used to avoid cross-contamination. During irrigation, the position of the patient was changed from Trendelenburg position to reversed Trendelenburg position and vice versa, until each position was adopted twice.

### 2.3. Sample Preparation

Cell blocks were prepared from individual peritoneal washings. Briefly, citrate plasma and thromboplastin were added after centrifugation, and the resulting cellular pellet was embedded in paraffin. Immunostaining using a smooth-muscle actin antibody (Ventana Medical Systems, Tucson, AZ, USA) was performed with a Benchmark Ultra autostainer (Ventana Medical Systems, Tucson, AZ, USA) following in-house validated protocols using an incubation time of 32 min at 36 °C [12]. For visualization of antigen–antibody binding, the ultraView Universal DAB detection kit (Ventana/Roche) was used, which contains a cocktail of horseradish peroxidase (HRP)-labeled antibodies (goat anti-mouse IgG, goat anti-mouse IgM, and goat anti-rabbit).

The samples were analyzed by a specialized gynecologic pathologist. They were considered “positive” in the presence of at least one smooth muscle cell.

### 2.4. Statistical Analysis

Statistical analysis was conducted with R [13] and packages Barnard [14], ggplot2 [15], ggsurvfit [16], samplesizeCMH [17], and survival [18]. Numerical data are presented via medians as well as the first and the third quartile. Categorical data are described via absolute frequencies. In order to examine such data, we used the Cochran–Mantel–Haenszel test, followed by Barnard’s test. For survival analysis, Kaplan–Meier estimators were calculated. *p*-Values < 0.05 were considered statistically significant. For the Cochran–Mantel–Haenszel test, under the assumptions of a significance level of 0.05 and an overall sample size of 275 (=number of observations across all strata), we computed a power of 1. For the analyses regarding cell spread, 11 false-negative irrigation samples (detection of cell spread in irrigation 5, though no detection of cell spread in irrigation 3 or 4) were converted into positive samples. As post hoc analysis revealed no significant differences in cell spread between Groups B, C, and D, these groups were combined (B + C + D) for comparative analysis against Group A at later timepoints in order to facilitate an easier comparison between myomectomy with power morcellation (Group A) and TLH groups.

### 2.5. AI Statement

During the preparation of this manuscript, the authors used ChatGPT 3.5 in order to improve readability. After using this tool, the authors reviewed and edited the content as needed and take full responsibility for the content of the publication.

## 3. Results

In total, 409 samples of 72 patients were analyzed. Two patients were excluded after surgery, one because of intraoperative anesthesiologic complications and non-completion of the procedure and one because of conversion to laparotomy (see Supplementary Figure S1). Patient characteristics and differences between the study groups are provided in Table 1. Testing of multiple comparisons confirmed a significantly lower age, gravidity, and parity (*p* < 0.001) for women undergoing myomectomy and power morcellation (Group A) compared to the hysterectomy groups (B, C, and D). There were no significant differences in these characteristics between Groups B, C, and D.

**Figure 1:**
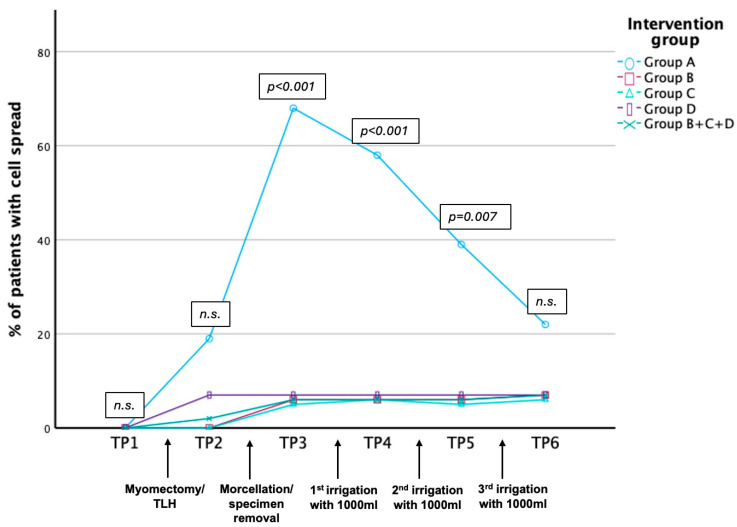
Summary of patient percentage with detected cell spread for every timepoint (TP) of peritoneal washing. TP 1 (before surgery), TP 2 (after myomectomy/TLH), TP 3 (after morcellation; =after specimen removal if morcellation was not applied), TP 4 (after irrigation with 1000 mL), TP 5 (after irrigation with 2000 mL), and TP 6 (after irrigation with 3000 mL) in different groups; Group A, myomectomy and power morcellation; Group B, TLH with en bloc transvaginal tissue removal without morcellation; Group C, TLH with manual vaginal morcellation; Group D, TLH with contained manual vaginal morcellation using a contained extraction system. TLH Groups B + C + D were combined post hoc based on statistical similarity. TLH, total laparoscopic hysterectomy.

Cell spread in peritoneal washings for every timepoint (TP) in the study groups is shown in Figure 1/Supplementary Table S1. All peritoneal washings taken prior to hysterectomy/myomectomy (TP1) were negative. A total of 23 (5%) peritoneal washings were inadvertently not performed (10 in Group A, 4 in Group B, 5 in Group C, and 4 in Group D) (Supplementary Figure S1).

Smooth muscle cells were found in four patients (4/21 19%) after myomectomy (Group A) and in one patient (1/48, 2%) after hysterectomy (Groups B, C, and D) (*p* = *0*.029) before morcellation (TP2).

In Group A, immediately after power morcellation of myomectomy specimens (TP3), smooth muscle cells were detected in the peritoneal washings of 68% (13/19) of patients, as opposed to 6% (3/50) in the TLH groups [1/17 (6%) in Group B (TLH alone), 1/19 (5%) in Group C (TLH with uncontained vaginal morcellation), 1/14 (7%) in Group D (TLH with contained vaginal morcellation); *p* < 0.001]. There was no difference between Groups B, C, and D (1/17 (6%), 1/19 (5%) and 1/14 (7%), respectively). Therefore, Groups B, C, and D were merged for further comparisons after this timepoint.

**Figure 2:**
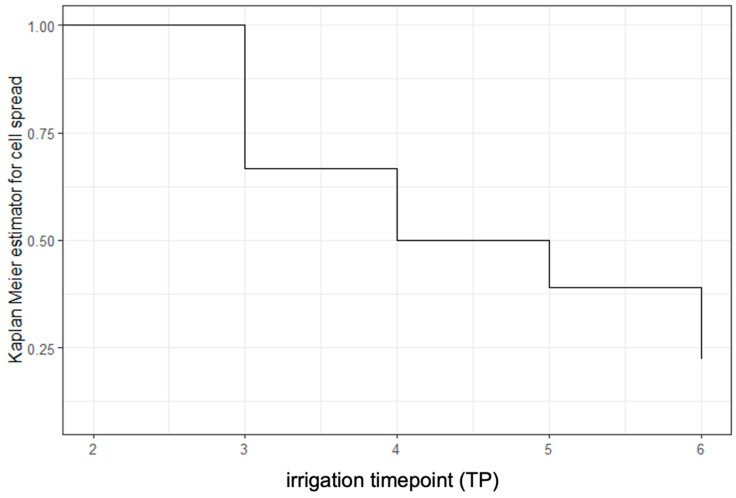
Kaplan–Meier curve representing survival of cell spread of smooth muscle cells in peritoneal washings.

The percentage of positive washings in Group A declined with increasing amounts of irrigation [58% (11/19) after 1000 mL and 39% (7/18) after 2000 mL], but it remained significantly elevated in comparison to the merged Groups B, C, and D at these timepoints (TP4&5) (*p* < 0.001 and *p* = *0*.007). After abdominal irrigation with a total of 3000 mL physiological saline solution (TP6), there was no more significant difference between the groups [4/18 (22%) versus 3/45 (7%), *p* = 0.079]. Survival analysis confirmed a continuous decrease in smooth muscle cells found in peritoneal washings after every 1000 mL of irrigation (Figure 2).

Pathohistologic analysis results are provided in Supplementary Table S2. Uterine fibroids were present in 61 patients (85%) and adenomyosis in 16 patients (22%). Furthermore, one patient had an occult uterine leiomyosarcoma (1%, Group C), two patients had an occult smooth muscle tumor of uncertain malignant potential (3%, 1 in Group A and 1 in Group D), and one patient had an occult endometrial adenocarcinoma within an adenomyosis lesion (1%, Group C).

## 4. Discussion

Following the FDA’s safety warning against power morcellators in laparoscopic myomectomy and hysterectomy, the use of minimally invasive approaches has significantly declined [3]. This decline is driven by hospital mandates and concerns about legal repercussions [19], highlighting the risk of worsening oncological outcomes due to the intraoperative spread of occult malignancies, especially LMS [20,21,22,23,24]. This cautious approach often overshadows the proven benefits of minimally invasive surgery compared to open surgery. However, questions remain about the impact of different morcellation techniques on oncological outcomes.

Furthermore, aside from the potential oncological consequences, there are non-malignant risks such as parasitic fibroid formation, which occurs in 0.12% to 0.95% of cases [25]. Additionally, morcellation of the uterus carries the risk of scattering endometrial cells, which could give rise to the potential development of endometriosis following uterine morcellation [26]. In a study analyzing 279 patients undergoing a second laparoscopy after supracervical hysterectomy with unconfined power morcellation, 23.3% were diagnosed with de novo endometriosis, which prompts concerns about a potential link between morcellation and the development of endometriosis [27].

In the past decade, various techniques and devices have been developed to reduce cell spread during morcellation, aiming to preserve the advantages of minimally invasive surgery [28,29,30].

Our study was designed to investigate whether irrigation after morcellation could reduce or eliminate cell spread. We also sought to analyze the effect of different morcellation techniques, particularly recognizing that the risks associated with power morcellation may not apply to transvaginal cold knife morcellation. To avoid bias, we randomized patients undergoing cold knife morcellation to either in-bag or uncontained morcellation.

Our study found that irrigation with 3000 mL of saline led to a continuous decrease in smooth muscle cells in peritoneal washings, reaching levels comparable to those observed after en bloc TLH. These findings align with those of Yu et al. [11] Cell spread was significantly more common in patients undergoing laparoscopic myomectomy with power morcellation compared to those undergoing TLH with transvaginal cold knife morcellation, whether contained or uncontained. Notably, 19% of myomectomy patients showed cell spread already before power morcellation compared to only 2% after hysterectomy, which confirms previous studies postulating that cell spread occurs already after fibroid enucleation and before power morcellation [9,31]. These results suggest that simply avoiding power morcellation by removing the intact fibroid through an abdominal incision does not necessarily protect against cell spread and potential sequelae. Additionally, myometrial cells were found even after hysterectomy without morcellation, indicating that cell spread cannot be entirely prevented, even with en bloc hysterectomy. Therefore, our findings do not claim to eliminate oncologic risk, but aim to contribute in efforts to reduce the cell spread associated with laparoscopic surgery, which may be particularly important for mitigating non-oncologic complications (e.g., parasitic leiomyomas, iatrogenic endometriosis).

Our study has limitations, notably the lack of a prospective sample size calculation due to its pilot nature. The non-significant differences observed may be due to the small patient cohort. This may apply especially to our finding that after irrigation with 3000 mL of saline, there was no significant difference in cell spread between the myomectomy with power morcellation group and the hysterectomy groups with or without transvaginal cold knife morcellation. Nevertheless, our data show a continuous decrease in cell spread with increased irrigation, and a post hoc power analysis, with a power of 1, indicates that the sample size for our statistical analysis is sufficient. Furthermore, we did not explore variations in the timing, volume distribution, or dwell time of irrigation. It remains unclear whether a single larger-volume irrigation or prolonged exposure might yield similar or superior results. In light of the unexpectedly high rate of occult malignancy in our cohort (4/72), we also acknowledge the limitations of the current preoperative diagnostic protocols. While all patients underwent examination and imaging by experienced gynecologists, these findings underscore the challenges of completely excluding malignancy preoperatively. However, this observation is consistent with recent literature emphasizing the need for improved imaging strategies in evaluating uterine masses [32] and aligns with current guidelines for managing uterine sarcomas [33]. It is worth mentioning here that the lack of long-term follow-up data in this pilot study, together with the limited sample size, prevents assessment of recurrence rates or clinical outcomes related to residual cell spread. Another limitation is that in peritoneal washings, smooth muscle cells appear as single cells or small clusters with often significant thermal and mechanical alterations, making it difficult to determine their origin. Furthermore, the spread of smooth muscle cells during benign procedures can only be a surrogate for cell spread in cases of occult LMS, as malignant cells possess distinct biological properties. Therefore, our study should be interpreted as a step towards reducing avoidable cell dissemination in benign surgeries, rather than as a substitute for robust preoperative oncologic risk assessment.

Future studies with longer follow-up and larger cohorts are needed to confirm whether the investigated strategies aiming to minimize cell and tissue remnants after surgical procedures translate into lower rates of long-term complications or into better long-term outcomes. Future research should also pay particular attention to cold knife morcellation and subsequent irrigation, contained morcellation, irrigation protocols, and the combination of these techniques, as this holds promise for preserving minimally invasive options in complex benign gynecological surgeries and incremented safety in the context of undiagnosed malignancies.

## 5. Conclusions

This prospective pilot study investigated peritoneal cell dissemination during benign laparoscopic uterine surgeries at different timepoints. We confirmed that cell spread occurs not only during morcellation, emphasizing that cell dissemination can begin before tissue fragmentation. Notably, transvaginal cold knife morcellation was associated with significantly lower rates of peritoneal cell spread compared to laparoscopic power morcellation. Copious peritoneal irrigation with 3000 mL of saline consistently reduced the cell count, ultimately lowering residual cell presence in myomectomy with power morcellation to levels comparable to those seen in en bloc hysterectomy. Given its simplicity, safety, and compatibility with standard minimally invasive techniques, we recommend incorporating copious irrigation as a routine adjunct during myomectomy or hysterectomy procedures involving morcellation. Furthermore, we believe that our findings may also support preoperative counseling and help preserve the advantages of minimally invasive surgery, even in cases involving large specimens that require vaginal morcellation.

## Figures and Tables

**Table 1:** Baseline characteristics of the study groups.

Variable	All	Group A	Group B	Group C	Group D	Group B + C + D	*p*-Value A vs. B + C + D *
*n*	72	21	17	19	15	51	
Age (years)	43 (38–45)	37 (33–40)	43 (41–45)	44 (41–46)	47 (43–51)	44 (41–47)	*p* < 0.001
BMI (kg/m^2^)	25 (23–31)	27 (22–26)	24 (23–31)	26 (25–30)	23.5 (22–25)	25 (23–29)	*p* = 0.451
Gravidity	2 (1–3)	1 (0–1)	2 (0–4)	2 (2–3)	2 (1–2)	2 (1–3)	*p* < 0.001
Parity	1 (0–2)	0 (0–1)	2 (0–2)	2 (1–2)	2 (1–2)	2 (1–2)	*p* < 0.001

Group A, myomectomy and power morcellation; Group B, TLH without morcellation; Group C, TLH with manual vaginal morcellation; Group D, TLH with contained manual vaginal morcellation using a contained extraction system; Group B + C + D, TLH; * Kruskal–Wallis rank sum test, *p*-value comparing Group A to Groups B + C + D.

## Data Availability

The raw data supporting the conclusions of this article will be made available by the authors on request.

## Supplementary Materials

The following supporting information can be downloaded at https://www.mdpi.com/article/10.3390/jcm14103383/s1: Supplementary Table S1: Summary of samples with detected cell spread and samples available for every timepoint (TP) of peritoneal washing. Supplementary Table S2: Pathohistological analysis results. Supplementary Figure S1: Sample flowchart.

## Abbreviations

The following abbreviations are used in this manuscript:

LM	Laparoscopic myomectomy
TLH	Total laparoscopic hysterectomy
TP	Timepoint
ml	Milliliters
FDA	U.S. Food and Drug Administration
CRF	Case Report Form
cc	Cubic centimeter
BMI	Body mass index
(u)LMS	(Uterine) leiomyosacroma
min	Minimum
max	Maximum
cm	Centimeter
STUMP	Smooth muscle tumor of uncertain malignant potential
FIGO	The International Federation of Gynecology and Obstetrics
