# Supplementary material for: Reducing Peritoneal Cell Dissemination in Laparoscopic Uterine Surgery: A Comparative Pilot Study on Morcellation Techniques and Peritoneal Irrigation
Source: J Clin Med. 2025 May 13;14(10):3383. doi: 10.3390/jcm14103383 (PMC12112552; doi:10.3390/jcm14103383)
Supplement: Supplementary file 1 [file jcm-14-03383-s001.zip › Table S1.pdf]

Table S1. *Summary of samples with detected cell spread and samples available for every timepoint (TP) of peritoneal washing.*

Irrigation Timepoints	Group A (N=21)	Group B (N=17)	Group C (N=19)	Group D (N=15)	B+C+D (N=51)	A vs B+C+D*
TP1	0/21	0/17	0/19	0/15	0/51	p=1
TP2	4/21 (19%)	0/16	0/17	1/15 (7%)	1/48 (2%)	p=.029
TP3	13/19 (68%)	1/17 (6%)	1/19 (5%)	1/14 (7%)	3/50 (6%)	p<.001
TP4	11/19 (58%)	1/17 (6%)	1/18 (6%)	1/14 (7%)	3/50 (6%)	p<.001
TP5	7/18 (39%)	1/17 (6%)	1/19 (5%)	1/14 (7%)	3/50 (6%)	p=.007
TP6	4/18 (22%)	1/14 (7%)	1/17 (6%)	1/14 (7%)	3/45 (7%)	p=.079

TP 1 (before surgery), TP 2 (after myomectomy/TLH), TP 3 (after morcellation; = after specimen removal if morcellation was not applied), TP 4 (after irrigation with 1000ml), TP 5 (after irrigation with 2000ml) and TP 6 (after irrigation with 3000ml) in different groups; Comparison of cell spread in GROUP A vs. GROUP B+C+D. Missing samples are shown in Supp. Figure 1; GROUP A, myomectomy and power morcellation; GROUP B, TLH with en bloc transvaginal tissue removal without morcellation*; GROUP C, TLH with manual vaginal morcellation; GROUP D, TLH with contained manual vaginal morcellation using a contained extraction system. TLH total laproscopic hysterectomy *Barnard´s exact test*
