# Supplementary material for: Reducing Peritoneal Cell Dissemination in Laparoscopic Uterine Surgery: A Comparative Pilot Study on Morcellation Techniques and Peritoneal Irrigation
Source: J Clin Med. 2025 May 13;14(10):3383. doi: 10.3390/jcm14103383 (PMC12112552; doi:10.3390/jcm14103383)
Supplement: Supplementary file 1 [file jcm-14-03383-s001.zip › Table S2.pdf]

Table S2. Pathohistological analysis results.

Variable	All	Group A	Group B	Group C	Group D
number of fibroids, median (min - max)	1 (1-10)	1 (1-10)	1 (1-3)	1 (1-2)	1 (1-3)
size of main fibroid (cm), median (min-max)	6 (1-12)	6 (2-10)	5 (2-7)	6 (4-12)	5 (1-9)
final histology, number (%)					
fibroids	61 (85%)	20 (95%)	14 (88%)	16 (84%)	12 (8%)
adenomyosis	16 (22%)	0	4 (25%)	8 (42%)	4 (27%)
uLMS	1 (1%)	0	0	1 (5%)	0
STUMP	2 (3%)	1 (5%)	0	0	1 (7%)
endometrial adenocarcinoma	1 (1%)	0	0	1 (5%)	0

GROUP A, myomectomy and power morcellation; GROUP B, TLH with en bloc transvaginal tissue removal without morcellation; GROUP C, TLH with manual vaginal morcellation; GROUP D, TLH with contained manual vaginal morcellation using a contained extraction system; min, minimum; max, maximum; FIGO, The International Federation of Gynecology and Obstetrics; uLMS, uterine leiomyosarcoma; STUMP, smooth muscle tumor of uncertain malignant potential;
