# Supplementary figures and images for: Reducing Peritoneal Cell Dissemination in Laparoscopic Uterine Surgery: A Comparative Pilot Study on Morcellation Techniques and Peritoneal Irrigation
Source: J Clin Med. 2025 May 13;14(10):3383. doi: 10.3390/jcm14103383 (PMC12112552; doi:10.3390/jcm14103383)

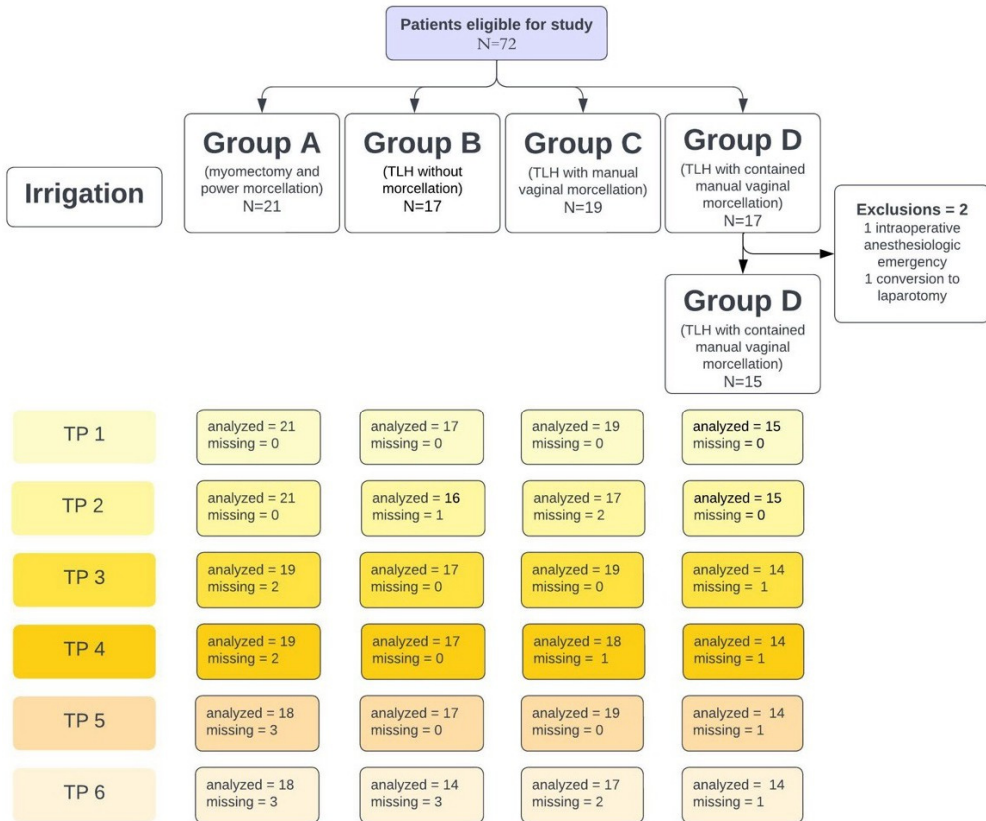

Supplement: Supplementary file 1 [file jcm-14-03383-s001.zip › Figure S1.pdf]
